# Evaluation of Risk Prediction Models to Identify Cancer Therapeutics Related Cardiac Dysfunction in Women with HER2+ Breast Cancer

**DOI:** 10.3390/jcm11030847

**Published:** 2022-02-05

**Authors:** Sivisan Suntheralingam, Chun-Po Steve Fan, Oscar Calvillo-Argüelles, Husam Abdel-Qadir, Eitan Amir, Paaladinesh Thavendiranathan

**Affiliations:** 1 Ted Rogers Program in Cardiotoxicity Prevention, Peter Munk Cardiac Center, Toronto General Hospital, University Health Network, University of Toronto, Toronto, ON M5G 2N2, Canada; sivisan.suntheralingam@mail.utoronto.ca (S.S.); ocalvillo@hsnsudbury.ca (O.C.-A.); husam.abdel-qadir@wchospital.ca (H.A.-Q.); 2 Rogers Computational Program, Ted Rogers Centre for Heart Research, Peter Munk Cardiac Centre, University Health Network, Toronto, ON M5G 2N2, Canada; s.fan@uhnresearch.ca; 3 Department of Cardiology, Department of Medical Oncology, Health Sciences North (HSN), Division of Clinical Sciences, Northern Ontario School of Medicine (NOSM), Sudbury, ON P3E 2C6, Canada; 4 Institute of Health Policy Management and Evaluation, University of Toronto, Toronto, ON M5T 3M6, Canada; 5 Women’s College Hospital (WCH), Toronto, ON M5S 1B2, Canada; 6 Division of Medical Oncology and Hematology, Princess Margaret Cancer Centre, University Health Network, Toronto, ON M5G 2C1, Canada; eitan.amir@uhn.ca

**Keywords:** cardiotoxicity, risk prediction models, cardiac dysfunction, trastuzumab-induced cardiotoxicity

## Abstract

Cancer-therapeutics-related cardiac dysfunction (CTRCD) is an important concern in women receiving trastuzumab therapy for HER2+ breast cancer. However, the ability to assess CTRCD risk remains limited. In this retrospective cohort study, we apply three published risk prediction models (Ezaz et al., NSABP-31 cardiac risk scores (CRS), and HFA-ICOS trastuzumab proforma) to 629 women (mean age 52.4 ± 10.9 years) with Stage I-III HER2+ breast cancer treated with trastuzumab ± anthracyclines to assess their performance to identify CTRCD during or immediately post treatment. Using these models, patients were classified into CTRCD risk categories according to the pre-treatment characteristics. With NSABP-31 CRS and HFA-ICOS proformas, patients in the highest risk category had a 1.7-to-2.4-fold higher relative risk of CTRCD than the low-risk category (*p* = 0.010 and 0.005, respectively). However, with all three risk models, those in the low-risk category had a high absolute risk of CTRCD (15.5–25.5%). The discrimination of the models for CTRCD (AUC 0.51–0.60) and their calibration was limited. NSAP-31 CRS and HFA-ICOS proformas can identify relative differences in CTRCD risk between patients, but when considering absolute risk, they are only able to identify the highest risk patients. There remains an ongoing need for accurate CTRCD risk prediction models in women with HER2+ breast cancer.

## 1. Introduction

Cancer-therapeutics-related cardiac dysfunction (CTRCD) can occur in >15% of women receiving anthracycline and trastuzumab therapy for HER2+ breast cancer [1,2,3]. This risk may be increased in the presence of cardiovascular risk factors [4,5]. The development of CTRCD may herald an increased risk of heart failure. Moreover, CTRCD is among the most common causes for HER2 therapy interruption, which in turn may be associated with a worse recurrence risk [6,7]. As such, cardiovascular risk management is a priority within the integral care of this population.

While risk factors for CTRCD development are well recognized, our ability to accurately determine patient-specific CTRCD risk remains limited [8,9]. Two different risk models have been published with the aim of CTRCD risk prediction in trastuzumab treated patients both during and after treatment [10,11]. However, these risk models have limited [12] or no external validation. More recently, the Heart Failure Association (HFA) of the European Society of Cardiology in collaboration with the International Cardio-Oncology Society (ICOS) developed proformas that proposed an expert-opinion-based weighted model with the intention of facilitating pre-cancer therapy risk stratification and cardio-oncology referral [13]. This model has also had limited validation [14].

In order to use these models to identify CTRCD risk during treatment, it is important to establish whether these models work in populations outside those in whom they were derived, or in the case of HFA-ICOS proformas, to validate what is considered expert opinion. In this study, we seek to assess the performance of these models for the identification of CTRCD during cancer treatment by determining the discrimination and calibration of these three models in a cohort of women with HER2+ breast cancer who received treatment with chemotherapy and trastuzumab in routine clinical practice.

## 2. Materials and Methods

### 2.1. Patients

In this retrospective cohort study, we included women ≥18 years of age with HER2/neu over-expressing (HER2+) breast cancer (stage I-III) treated with trastuzumab with or without anthracycline at the Princess Margaret Cancer Centre, Toronto, Canada, between 2006 and 2019. We included patients who had a pre-therapy multi-gated acquisition (MUGA) scan or 2D echocardiogram and ≥3 follow up scans. Women with stage IV cancer and those who were deemed palliative were excluded from this study. For each patient, the following data were obtained through electronic patient records: demographics, previous cardiac history, cardiac medications at baseline, cardiac risk factors, baseline breast cancer characteristics, cancer treatment (including anthracycline dose, radiotherapy, surgery) and LVEF data. Patients were followed until the completion of the trastuzumab therapy.

### 2.2. Cancer Therapeutics Related Cardiac Dysfunction (CTRCD)

Our primary definition of CTRCD was based on the Cardiac Review and Evaluation Committee (CREC) criteria using the same imaging modality: an absolute LVEF decline of ≥10% to <55% without HF symptoms or ≥5% drop to <55% with HF symptoms at any point during or immediately post cancer therapy [15]. We also performed sensitivity analysis using the European Society of Cardiology (ESC) definition—an absolute reduction in LVEF >10% to a value below 50% [16]—and the American Society of Echocardiography (ASE) definition—an absolute reduction in LVEF >10% to below 53% [17]. For all definitions, when the baseline LVEF was below the lower limit of normal, an additional >10% reduction in LVEF was used to define CTRCD.

### 2.3. Risk Models

For each patient, we classified their CTRCD risk using the models developed by Romond et al. (National Surgical Adjuvant Breast and Bowel Project B-31 cardiac risk score (NSABP-31 CRS)) [11] and Ezaz et al. [10], and based on the HFA-ICOS trastuzumab proforma [13]. Please refer to Supplementary Materials Table S1 for details regarding the risk models. The model by Ezaz et al. (hereafter referred to as the Ezaz model), was developed with the Surveillance, Epidemiology and End Results (SEER)-Medicare database to predict heart failure/cardiomyopathy risk within 3 years of cancer diagnosis in older women with HER2+ breast cancer who received adjuvant trastuzumab therapy. For this model, patient and treatment characteristics were associated with numerical points, with total points ranging from 0 (lowest risk) to 12 (highest risk). The authors, however, only reported CTRCD incidence after aggregating the points into three categories (i.e., 0–3 as low risk; 4–5 as medium risk; 6–9 as high risk). The CTRCD outcome for the Ezaz model (heart failure or cardiomyopathy) was based on ICD-9 codes.

The NSABP-31 CRS was developed using part of the NSABP-31 clinical trial cohort and utilized baseline LVEF and age to calculate a score (Supplementary Materials Table S1) in women with HER2+ breast cancer treated with anthracyclines and trastuzumab [11]. These patients were followed for up to a total of 7 years. The NSABP-31 CRS predicted the probability of “definite or probable cardiac death or congestive heart failure (CHF) manifested by dyspnea with normal activity or at rest and associated with an absolute decrease in LVEF of greater than 10 percentage points from baseline to a value less than 55% or a decrease of more than 5% to a value below the lower limit of normal”. The majority of the cardiac outcomes in this study (95%) occurred within the first 2 years [18].

With the HFA-ICOS proformas for trastuzumab (hereafter referred to as HFA-ICOS proforma), patient and treatment factors are designated from medium-to-very-high risk [13]. After the risk factors are identified, a final risk category combining these risk factors is derived (Supplementary Materials Table S1). This score was based on expert opinion with the suggested definition of risk categories for future CTRCD as follows: low <2%, medium 2–9%, high risk 10–19%, and very high >20%. Events defining CTRCD or their timing were not defined. The HFA-ICOS proforma also included optional consideration of biomarkers (troponin and B-type natriuretic peptide); however, given the retrospective nature of our study and lack of standardized biomarker collection, we did not have this to include for our full cohort; however, in the subgroup with patients that had these data available, we performed a subgroup analysis.

### 2.4. Statistical Analysis

Patient characteristics were reported using descriptive statistics. Continuous variables are summarized using the mean ± standard deviation. For the risk models, chi-square test or Fisher’s exact tests were used to assess statistical differences in the incidence of CTRCD between risk categories. One-way ANOVA was used to assess differences in continuous variables between the risk categories for the models.

We assessed the discriminatory accuracy of the risk models using the area under the receiver operating characteristic curves (AUC). We summarized the model calibration using calibration plots (i.e., predicted vs. observed absolute risk) for each model. The predicted CTRCD risks for the Ezaz model were based on the incidence of CTRCD described in the original publication for 3 risk categories (Supplementary Materials Table S1). For the HFA-ICOS proforma, the predicted CTRCD risk was based on the suggested risk for the four risk categories in the original publication. To calculate the predicted risk for the NSABP-31 CRS, knowledge of the cumulative incidence function in terms of CRS is required; however, the authors did not provide this information. The authors outlined the CRS formulation and visually presented the functional relation between CRS and the risk of cardiac events (see Figure 4A of Romond et al.) [11]. Therefore, we used the reported CRS and the risk of cardiac events in a subset of patients in the Supplementary Materials and approximated the functional relation between CRS and the risk of cardiac events using restricted cubic splines. Subsequently, we calculated the risk of cardiac events for the validation patients and stratified them based on the quintiles of the predicted risk given that the original study did not divide patients into risk categories. Moreover, to enable the comparison of the NSABP-31 CRS with the other scores in the exploratory analysis, we divided the cohort into a low, medium, and high-risk category based on the tertiles of the distribution of the score in our cohort. Finally, we calculated the proportion of patients with CTRCD in our cohort to be the observed risk in each risk group for each model. The 95% CIs were estimated using logistic regression.

Statistical tests were two-sided and *p* < 0.05 was considered to be statistically significant. All statistical analyses were performed with SPSS (*v*27, IBM Inc., Armonk, NY, USA) and R (*v*4.0.3, R Foundation for Statistical Computing, Vienna, Austria).

## 3. Results

### 3.1. Patients

A total of 629 women were included. The mean age was 52.4 ± 10.9 years and the baseline LVEF was 65 ± 6.7%. There were 151 (24%) patients who developed CTRCD. The baseline characteristics of the patients are provided in Table 1. Those who developed CTRCD had a lower baseline left ventricular ejection fraction (*p* < 0.001). There was a greater use of MUGA scans than echocardiograms in our patients given the time period over which patients were treated. Comparisons of patient characteristics for each model based on the various risk categories are provided in Supplementary Materials Table S2 and the available baseline characteristics from the original studies are presented in Supplementary Materials Table S3. As expected, with all the risk models, patients deemed to be at higher risk in our study were older and were more likely to have baseline cardiovascular risk factors.

### 3.2. Incidence of CTRCD within Risk Score Categories

When the Ezaz model was applied to our cohort, there were 577 (92%), 45 (7%), and 7 (1%) patients in the low-, medium- and high-risk categories of whom CTRCD was identified in 141 (24.4%), 9 (20%), and 1 (14.3%) patients, respectively (*p* = 0.67).

With the NSABP-31 CRS, based on the distribution of the score in our cohort, we classified patients into tertiles of CRS. There were 223 patients (36%), 204 (32%), and 202 (32%) patients in the low (CRS ≤ 50), medium (CRS 51–64), and high (CRS ≥ 65 points) categories of whom CTRCD was identified in 40 (17.9%), 50 (24.5%), and 61 (30.2%) patients, respectively (*p* = 0.01).

With the HFA-ICOS proforma, 193 (31%), 404 (64%), 27 (4%) and 5 (1%) patients were in the low-, medium-, high-, and very-high-risk categories with CTRCD identified in 30 (15.5%), 109 (26.9%), 10 (37.0%), and 2 (40.0%) patients, respectively (*p* = 0.005).

### 3.3. Discrimination and Calibration of the Risk Models

**Figure 1:**
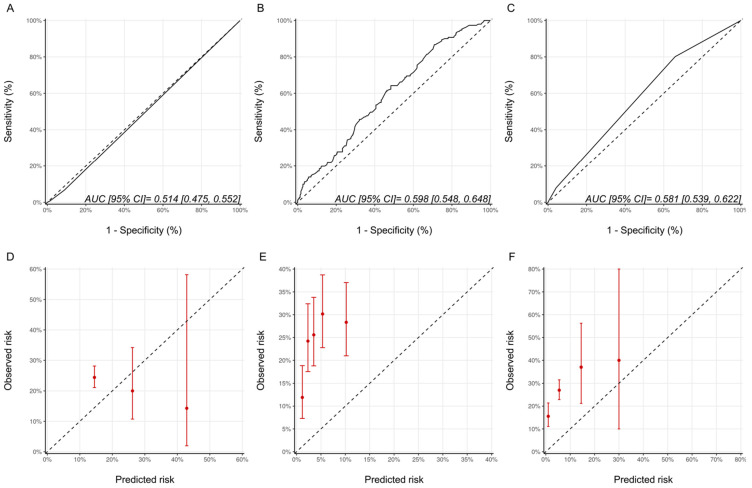
Assessment of discrimination and calibration for the three models. ROC Curve and Calibration plots for the Ezaz model (**A**,**D**). NSABP-31 CRS (**B**,**E**) and HFA-ICOS Proforma (**C**,**F**). In Panel A-C, the dotted line represents how a random classifier would perform while the solid line represents the performance of the model being evaluated. The AUCs and 95% CIs are provided as an insert. In Panel D-F, the dotted line represents perfect calibration. The red dots represent the observed risk of CTRCD of the risk groups, as defined by Ezaz model, the quintiles of NSABP-31 CRS, or HFA-ICOS Proforma, from our cohort. The red bars represent 95% CIs of the observed risks. NSABP-31 CRS, National Surgical Adjuvant Breast and Bowel Project B-31 cardiac risk score; HFA-ICOS Proforma, Heart Failure Association of the European Society of Cardiology-International Cardio-Oncology Society Proforma; AUC, area under curve; ROC Curve, receiver operating characteristic curves; CI, confidence interval.

As a measure of discrimination, receiver operator characteristic curves for the three models are presented in Figure 1. For the Ezaz model, AUC was 0.51 (95% CI: 0.48–0.55), NSABP-31 CRS 0.60 (95% CI: 0.55–0.65) and the HFA-ICOS proforma 0.58 (95% CI: 0.54–0.62).

The calibration plots for all the models are shown in Figure 1 and were generally poor across all three models. For the Ezaz model, the low-risk group had the highest observed CTRCD risk, and the high-risk group had the lowest observed risk. The estimated risks by NSABP-31 CRS (from the original publication) were consistently and substantially lower than the observed risk. HFA-ICOS Proformas also underestimated the risk considerably. The small number of patients in the higher risk categories of the Ezaz model and HFA-ICOS proforma contributed to the wide 95% CIs of the observed risks.

### 3.4. Sensitivity Analyses

**Figure 2:**
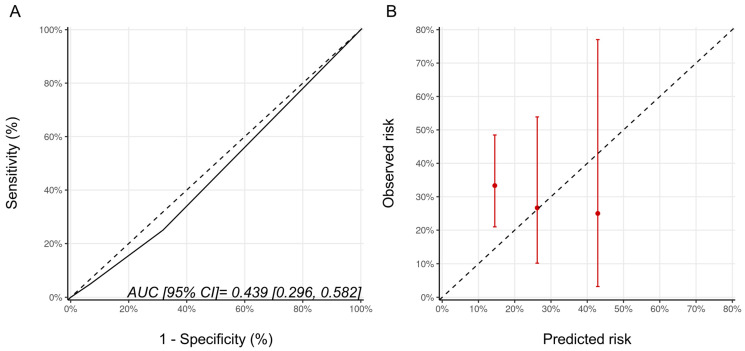
(**A**) ROC curve and (**B**) calibration plots for the Ezaz model for patients aged 67 years of age and older only. Please see the caption of Figure 1 for additional information regarding the figure. AUC, area under curve; ROC Curve, receiver operating characteristic curves.

As Ezaz et al. developed their model in women 67 years of age and older, we repeated the analysis only in these older patients from our cohort (*n* = 64). There were 45, 15, and 4 patients with low-, medium-, and high-risk categories of whom 15 (33.3%), 4 (26.7%), and 1 patient (25%), respectively, developed CTRCD (*p* = 0.856). Despite this alignment of the model development and validation cohorts, the model discrimination was poor (AUC (95% CI) 0.44 [0.30–0.58]), and the calibration remained suboptimal (Figure 2). The wide 95% confidence intervals reflect the small sample size of this subgroup.

**Figure 3:**
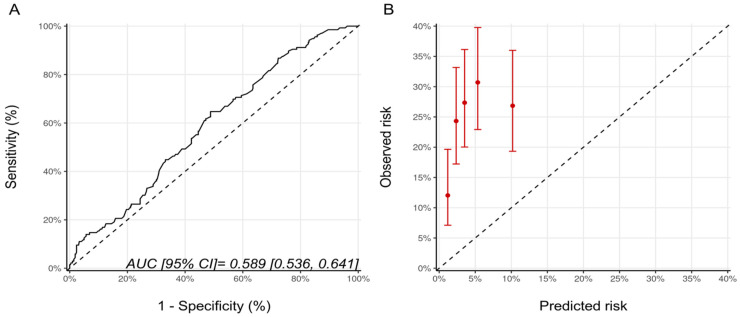
(**A**) ROC curve and (**B**) calibration plot for the NSABP-31 CRS with the inclusion, only, of patients who received sequential anthracycline and trastuzumab therapy. Please see the caption of Figure 1 for additional information regarding the figure. NSABP31-CRS, National Surgical Adjuvant Breast and Bowel Project B-31 cardiac risk score; AUC, area under curve; ROC Curve, receiver operating characteristic curves.

Since patients in the NSABP-31 study all received anthracycline, we repeated the analysis for the NSABP-31 CRS, including only the patients who received anthracyclines prior to trastuzumab (*n* = 568). There were 201, 184, and 183 patients, in the low, medium and high CRS categories with CTRCD diagnosed in 38 (18.9%), 47 (25.5%), and 53 (28.9%) patients, respectively (*p* = 0.064). The AUC for the score’s discrimination was unchanged compared to the whole cohort at 0.59 (95% CI: 0.54–0.64) and, overall, the estimated risks were lower than the observed risk of CTRCD (Figure 3).

**Figure 4:**
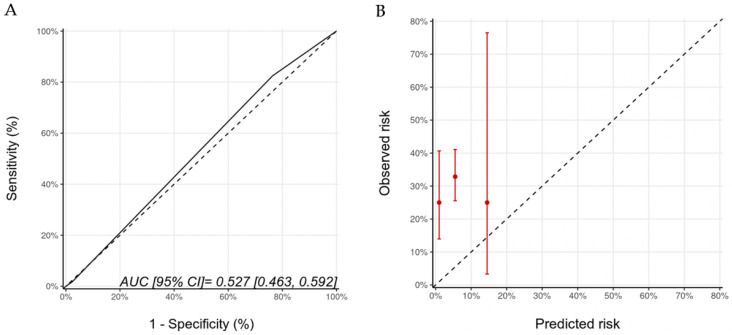
Discrimination (**A**) and calibration (**B**) of the HFA-ICOS proforma for patients with biomarkers (troponin and B-type natriuretic peptide). Please see the caption of Figure 1 for additional information regarding the figure. HFA-ICOS Proforma, Heart Failure Association of the European Society of Cardiology-International Cardio-Oncology Society Proforma; AUC, area under curve; ROC Curve, receiver operating characteristic curves.

Biomarkers were optional in the HFA-ICOS proformas. We examined the potential added value of baseline (pre-trastuzumab) troponin and BNP to the HFA-ICOS proformas. These measurements were available in 184 patients in our cohort; there were 26 patients who had pre-trastuzumab troponin and/or BNP that were above our institutional normal values (hsTnI > 16 ng/L, BNP > 100 pg/mL). A total of 7 patients were re-classified from the low to the medium risk category, 18 patients remained within the medium risk category, and 1 patient moved from the medium to the high-risk category. With the inclusion of biomarkers, there were 40 (22%), 140 (76%), and 4 (2%) patients in the low-, medium-, and high-risk categories of whom 10 (25%), 46 (33%), and 1 (25%) developed CTRCD, respectively (*p* = 0.617). There were no patients in the very-high-risk category. The AUC for discrimination was 0.53 (95% CI: 0.46–0.59) and the calibration remained poor (Figure 4). The wide 95% CI for the high-risk group reflects the small sample size in that group.

**Figure 5:**
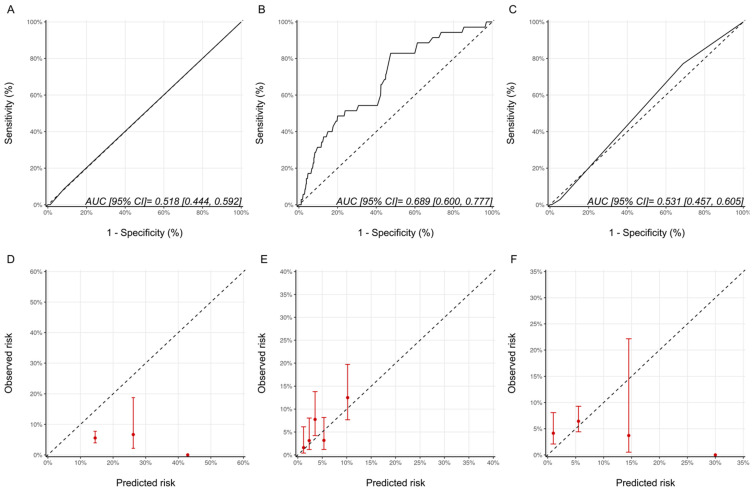
Discrimination and calibration of the risk models using the ESC CTRCD definition. ROC curves and calibration plots for the Ezaz model (**A**,**D**), NSABP-31 CRS (**B**,**E**), and HFA-ICOS proformas (**C**,**F**). Please see the caption of Figure 1 for additional information regarding the figure. ESC, European Society of Cardiology; NSABP31-CRS, National Surgical Adjuvant Breast and Bowel Project B-31 cardiac risk score; HFA-ICOS Proforma, Heart Failure Association of the European Society of Cardiology-International Cardio-Oncology Society Proforma; AUC, area under curve; ROC Curve, receiver operating characteristic curves.

Using the ESC and ASE definitions, 35 (5.6%) and 102 (16.2%) patients, respectively, in our cohort were classified as having CTRCD. The characteristics of patients with and without CTRCD based on these definitions are provided in Supplementary Materials Tables S4 and S5. The proportion of patients experiencing CTRCD with the ASE and ESC definitions of CTRCD in the various risk categories are summarized in Supplementary Materials Table S6. A statistically significant graded increase in CTRCD risk with increasing risk category was only seen with the HFA-ICOS proforma with the ASE definition and the NSABP-31 CRS with the ESC definition. With respect to discrimination, the AUCs for the Ezaz model, NSABP-31 CRS and HFA-ICOS proformas were 0.52 (95% CI: 0.44–0.59), 0.69 (95% CI: 0.60–0.78) and 0.53 (95% CI: 0.46–0.61), respectively (Figure 5). Compared to the CREC CTRCD definition, with the ESC definition, the NSABP-31 CRS showed better calibration, while there was an underestimation of risk with the Ezaz model for all risk categories and a better calibration at the lower risk categories for the HFA-ICOS proformas (Figure 5).

**Figure 6:**
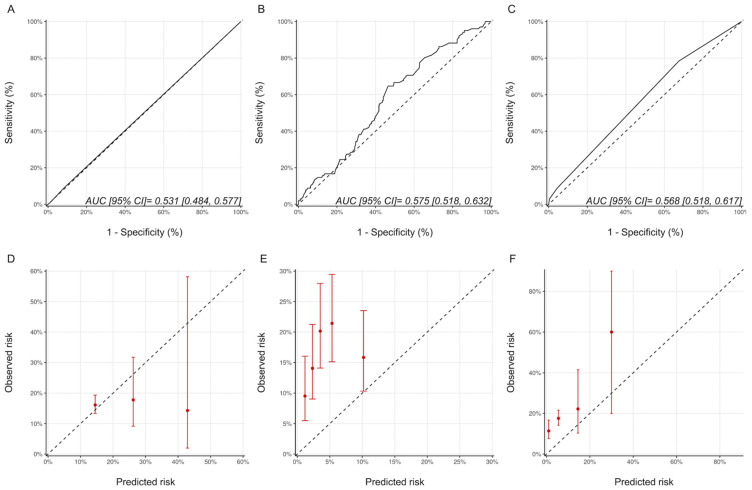
Discrimination and calibration of the risk modes using the ASE CTRCD definition. ROC curves and calibration plots for the Ezaz model (**A**,**D**), NSABP-31 CRS (**B**,**E**), and HFA-ICOS proformas (**C**,**F**). Please see the caption of Figure 1 for additional information regarding the figure. ASE, American Society of Echocardiography; NSABP31-CRS, National Surgical Adjuvant Breast and Bowel Project B-31 cardiac risk score; HFA-ICOS Proforma, Heart Failure Association of the European Society of Cardiology-International Cardio-Oncology Society Proforma; AUC, area under curve; ROC Curve, receiver operating characteristic curves.

For the ASE defined CTRCD, with respect to discrimination, the AUCs for the Ezaz model, NSABP-31 CRS and HFA-ICOS proformas were 0.53 (95% CI: 0.48–0.58), 0.58 (95% CI: 0.52–0.63) and 0.57 (95% CI:0.52–0.62), respectively (Figure 6). Compared with the CREC definition, the calibration for the Ezaz model was better; however, for the other two models, the calibration remained poor (Figure 6).

Finally, we assessed whether the use of MUGA versus 2D-echocardiography contributed to differences in CTRCD incidences for the various risk models and risk categories. Generally, a higher incidence of CTRCD was seen with 2D-echocardiography than MUGA (Supplementary Materials Table S7). Significant differences were seen in the medium risk category of the HFA-ICOS proforma (*p* = 0.021), the low-risk category for the Ezaz model (*p* = 0.021), and the low-risk category for the NSABP-31 CRS (*p* = 0.001).

## 4. Discussion

In this retrospective cohort study of women with HER2+ early-stage breast cancer treated with trastuzumab, we assessed the performance characteristics of three published risk models previously developed for the prediction of CTRCD. With the NSABP-31 CRS and the HFA-ICOS proforma, the incidence of CTRCD in our cohort increased with the higher risk categories, but this was not seen with the Ezaz model. With both the NSABP-31 CRS and HFA-ICOS proforma, patients deemed to be at a high or very high risk had a 1.7–2.4-fold higher incidence of CTRCD. However, the absolute CTRCD risk (as used in clinical practice) in the low-risk categories of all three risk models was high (15.5% to 25.5%), precluding their use to identify patients truly at low CTRCD risk (e.g., CTRCD incidence < 5%). Overall, the performance of all three models with respect to its discrimination for CTRCD and its calibration with published/suggested incidence was limited.

### 4.1. CTRCD Risk Assessment

Several risk factors for the development of CTRCD in women with HER2+ breast cancer receiving trastuzumab-based therapies have been described (e.g., age, low–normal LVEF, diabetes, hypertension, and anthracycline exposure) [4,9], but these risk factors in isolation are insufficient to accurately determine patient-specific CTRCD risk [8,9,16]. Unfortunately, cardiovascular disease risk prediction models used for the general population are not well suited for patients with cancer as they do not account for the competing risk for cancer death or the use of potentially cardiotoxic cancer therapy [19,20]. Therefore, cancer-specific risk models in these patients are much needed. These models can be used to help to guide cancer therapy (e.g., the use of anthracycline vs non-anthracycline based regimens) [21], support decisions regarding cardio-oncology consultation, escalated vs. de-escalated cardiac surveillance [4], initiation of cardio-protective therapy, and long-term cardiovascular follow-up. For example, less than 3% of imaging studies performed as part of routine screening in women with breast cancer have been shown to result in a change in care [22], suggesting that it may be possible to reduce the intensity of cardiac surveillance in a very large proportion of true low-risk individuals if we can identify them reliably. Furthermore, risk stratification may also enable the conduction of clinical trials of primary prevention using a risk-based as opposed to a “one-size-fits all” approach, as the benefits would most likely occur in higher risk patients [23].

### 4.2. Existing Risk Prediction Models

Given their potential importance, the development of CTRCD risk prediction models have remained a research priority in cardio-oncology. To date, there have been three published risk prediction models [10,11,13] for women with early stage HER2+ breast cancer, but these have had limited or no external validation. It is also of note that the HFA-ICOS proforma is an expert-consensus-derived risk model and hence has had limited validation [12]. To potentially promote more clinical uptake of these models, we sought to assess and compare the performance of these models in a large cohort of women with HER2+ breast cancer treated in a routine clinical practice. We focused on the diagnosis of CTRCD by the end of treatment, as this can affect ongoing cancer treatment and majority of CTRCD events in women with HER2+ early-stage breast cancer occur by the end of the treatment [4,11,18,24]. In our cohort, the NSABP-31 CRS and the HFA-ICOS proformas appear to identify patients with graded risk for CTRCD with the highest risk categories having a 1.7-to-2.4-fold higher risk of CTRCD compared to the lowest risk category. This was, however, not seen with the Ezaz model. An important finding in our cohort was that all three existing models were poor in identifying patients at low absolute risk of CTRCD as the risk in this group ranged from 15.5–24.4%. Given that majority of patients in clinical practice would not be at high risk for CTRCD, the inability to identify patients truly at low absolute risk of CTRCD is an important limitation of these existing models.

The Ezaz model had the poorest discrimination amongst the risk models with the lowest incidence of CTRCD seen in the highest risk group in our cohort. This may be due to several reasons. First, the majority of our patients (92%) fell into the low-risk category. The lowest risk patients in the Ezaz et al.’s derivation cohort were likely those who did not receive chemotherapy (16.4% of their cohort) or received non-anthracycline-based chemotherapy (47.7% of their cohort); however, only 10% of our cohort received non-anthracycline-based chemotherapy (Table 1). Secondly, we used a more sensitive definition of CTRCD, based on LVEF change as opposed to the ICD-9 based diagnosis of cardiomyopathy and HF. Thirdly, the Ezaz model does not consider baseline LVEF in risk calculation; however, this measure has consistently been identified as an important risk factor for CTRCD in trastuzumab-treated patients [11,25]. Fourthly, our follow-up duration was shorter (3 years versus ~1.5 years); however, most CTRCD events in these patients have been shown to occur by the end of the treatment [1,11,25]. Finally, Ezaz et al. included older patients (mean age 73.6 years vs. 52 years in our study) with greater cardiovascular co-morbidities (e.g., hypertension in 60% vs. 18% in our cohort). Our sensitivity analysis focusing on older patients did not show improved performance of the model. Overall, our findings are similar to a recent study that assessed the performance of the Ezaz model in patients referred to a cardio-oncology program where the incidence of CTRCD was 43%, 64%, and 30% in the low-, medium,- and high-risk groups, again suggesting the limited performance of the model [12].

When comparing our cohort to that of Romond et al.’s, their patients were slightly younger (mean age 49 vs. 52 years), but they had a similar prevalence of cardiovascular risk factors. There was a difference in CTRCD definition compared to our study as patients not only had to meet the LVEF change criteria (as in our study), but also have heart failure symptoms. Furthermore, CV death was included in their definitions, although only a single death was reported in the study. The detection of CTRCD was based on MUGA scans as was the case in the majority of our patients. Although their follow-up was up to 7 years, most cardiac events occurred by the end of therapy and is hence comparable to our cohort. The lower CTRCD risk predicted with this model compared to that seen in our study likely relates to the fact that (1) CTRCD required the presence of HF symptoms, which was not available in our study, and (2) patients in their study did not receive trastuzumab if there was a reduction in LVEF or cardiac symptoms post anthracyclines, but pre-trastuzumab (seen in 6.3% of their patients) potentially resulting in a lower CTRCD risk population compared to ours. (3) Finally, the study did not consider patients with asymptomatic declines in LVEF that required trastuzumab interruption in their risk model and this occurred in ~9% of their cohort.

The HFA-ICOS proforma for trastuzumab-treated patients was derived by expert consensus based on the existing literature on the importance of individual risk factors. This method had a similar discrimination to the NSABP-31 CRS with limited calibration, especially at the lower risk categories. These findings are similar to a recent study by Battisti et al., where the AUC for the HFA-ICOS proforma was reported to be 0.56 [14]. Therefore, there is a need to re-calibrate this model further for the identification of CTRCD. However, in our cohort, on balance, the HFA-ICOS proforma performed the best to identify CREC defined CTRCD. This is not surprising as it uses a combination of patient characteristics, treatment information, and cardiac function measures to estimate CTRCD risk.

### 4.3. CTRCD Definition and Model Performance

An important finding in the current and other recent studies [3] is the fact that the proportion of patients with CTRCD differed significantly based on the CTRCD definition used. These multiple CTRCD definitions have often affected the generalizability of a particular study’s findings in cardio-oncology. Therefore, for all three models, we considered the three accepted definitions of CTRCD (CREC, ASE, and ESC) as the outcome and re-assessed discrimination and calibration in the sensitivity analysis. With the ESC definition for all three models, the absolute risk of CTRCD in the low-risk group was more consistent with what would be expected clinically (2–6%); however, the number of events overall was small. With this definition, the discrimination of the NSABP-31 CRS (AUC 0.60 to 0.69) and the calibration for both the NSABP-31 CRS and the HFA-ICOS proformas improved. This suggests that perhaps the NSABP-31 CRS particularly may be better in identifying the absolute risk of more significant CTRCD (i.e., a fall in LVEF to <50%). With the ASE definition, there was no significant difference in discrimination or calibration for the three models compared to the CREC definition. This is not surprising given that the major difference between the CREC and ASE CTRCD criteria is the lower limit of normal LVEF of 55% versus 53%.

### 4.4. Limitations of Existing Models

Overall, there are several reasons why the current models may not have performed as well for the diagnosis of CTRCD in our cohort. This includes variability in CTRCD definition, exclusion of important cardiotoxicity-related outcomes, such as cessation of trastuzumab therapy for reduction in LVEF in model generation as in the NSABP-31 CRS, lack of inclusion of a comprehensive set of clinical variables (e.g., the use of cardiac medications was not included in any of the models), lack of use of dynamic clinical information (e.g., LVEF, strain, or biomarker change), and possibly absence of consideration of other risk factors, such as exercise, genomics and socioeconomic status. Perhaps with the incorporation of these components into risk models using data from large, well-characterized, multicenter cohorts, better CTRCD risk prediction models may be created.

### 4.5. Limitations

This is a retrospective cohort study and hence has its intrinsic limitations. Although we had a large cohort of patients, a larger cohort may have helped to better assess the discrimination and calibration of the models studied especially for the higher risk categories. For the risk models, there were some differences in the population in the original derivation cohort and our study. We performed a sensitivity analysis to address these differences whenever possible, showing that model performance did not change substantially overall. There were also differences in the period over which the CTRCD outcome was determined and the exact CTRCD definition in the NSABP-31 CRS, Ezaz models and our study. However, the existing literature suggests that the majority of CTRCD events in trastuzumab-treated patients occur by the end of the treatment period [1,11,25]. Furthermore, our focus on assessing the performance of the models to detect CTRCD during treatment is clinically important given that (1) this can result in cancer treatment interruption; (2) it is the period where there is the highest chance to promote ventricular function recovery; and (3) is the period where patients are most engaged with the health care system. Moreover, CTRCD during cancer treatment in these patients is prognostically important as it has been shown to be associated with long-term impact on cardiopulmonary fitness and persistent left ventricular dysfunction [26]. For the HFA-ICOS proformas, the outcome and the follow-up period were not pre-defined; however, we considered all three existing CTRCD definitions. Moreover, for the HFA-ICOS proformas, we chose 30% as the predicted probability for very high risk given the original manuscript suggested a risk of >20%; this may affect the visual assessment of model calibration. Finally, we had patients who were followed with echocardiograms and MUGA scans. Given that LVEF measurements with these modalities are not interchangeable, we ensured that we used the same modality to define CTRCD. Although 3D echocardiography is recommended as the method of choice to monitor for CTRCD and global longitudinal strain (GLS) used as a method to identify early cardiac injury, these measures were not universally available in our study given the recruitment period. However, our study was focused on comparing various CTRCD risk prediction models as opposed to examining methods to identify CTRCD.

## 5. Conclusions

Our study assessed performance characteristics of three published risk models previously developed for the prediction of CTRCD in a real-world clinical setting. Overall, the discrimination and calibration of the three models studied were limited in our cohort. With NSABP-31 CRS and the HFA-ICOS proforma, patients in the highest risk categories were at 1.7-to-2.4-fold higher risk of CTRCD by the end of the trastuzumab treatment compared to the low-risk patients. All three models were suboptimal in identifying a cohort with low absolute CTRCD risk. If used clinically, perhaps the role of these models may be in identifying patients at the highest absolute risk of CTRCD. Overall, on balance, amongst the three models, the HFA-ICOS proforma performed the best regardless of the CTRCD definition, while the NSABP-31 CRS performed the best with the ESC CTRCD definition. However, given the limitations of these models, there remains an ongoing need for robust risk prediction models to identify patients at risk of CTRCD during trastuzumab therapy. This would likely require a combination of clinical and genomic data, imaging measures, novel blood biomarkers, along with the ability to include early changes in these measures (i.e., a dynamic model) in order to accurately risk stratify patients for CTRCD. Such models are much anticipated in the field.

## Figures and Tables

**Table 1:** Summary of baseline characteristics of the whole cohort and stratified by CREC cardiotoxicity definition.

	All Patients N = 629	CTRCD N = 151	No CTRCD N = 478	*p* Value
Age, years ± SD	52.4 ± 10.9	52.7 ± 10.9	52.4 ± 10.9	0.827
Tumor laterality, *n* (%)				0.689
Left	341 (54)	79 (52)	262 (55)	
Right	268 (43)	68 (45)	200 (42)	
Bilateral	21 (3)	4 (3)	17 (4)	
Stage, *n* (%)				0.152
1	127 (20)	23 (15)	104 (22)	
2	329 (52)	80 (53)	249 (52)	
3	173 (28)	48 (32)	125 (26)	
Hormone receptor positive (either ER/PR), *n* (%)	413 (66)	98 (65)	315 (66)	0.821
Post-menopausal status, *n* (%)	309 (49)	72 (48)	237 (50)	0.709
Surgery (mastectomy or lumpectomy), *n* (%)	622 (99)	149 (99)	473 (99)	0.776
Anthracycline received, *n* (%)	568 (90)	138 (91)	430 (90)	0.604
Cumulative anthracycline, mg/m^2^ ± SD	272.4 ± 76	275.6 ± 80	260 ± 75	0.061
Radiotherapy, *n* (%)	493 (78)	127 (84)	366 (77)	0.499
Diabetes, *n* (%)	44 (7)	11 (7)	33 (7)	0.856
Hypertension, *n* (%)	110 (18)	30 (20)	80 (17)	0.391
Dyslipidemia, *n* (%)	83 (13)	25 (17)	58 (12)	0.169
Smoker (current), *n* (%)	46 (7)	13 (9)	33 (7)	0.476
Smoker (Ex), *n* (%)	122 (19)	31 (21)	91 (19)	0.723
At least 1 CVRF, *n* (%)	402 (64)	106 (70)	296 (62)	0.065
Coronary artery disease, *n* (%)	16 (3)	3 (2)	13 (3)	0.618
Heart failure, *n* (%)	5 (1)	1 (1)	4 (1)	0.833
Atrial fibrillation, *n* (%)	12 (2)	5 (3)	7 (2)	0.148
Baseline LVEF, % ± SD	64 ± 7	62 ± 7	65 ± 7	**0.001**
Imaging modality, *n* (%)				**0.010**
MUGA	440 (70)	93 (62)	347 (73)	
Echo	189 (30)	58 (38)	131 (27)	

CTRCD, cancer-therapeutics-related cardiac dysfunction; SD, standard deviation; ER, estrogen receptor; PR, progesterone receptor; CVRF, cardiovascular risk factor; CREC, cardiac review and evaluation committee; MUGA, multi-gated acquisition; Echo, echocardiography; LVEF, left ventricular ejection fraction.

## Data Availability

The data presented in this study are available on request from the corresponding author. The data are not publicly available due to patient confidentiality/privacy.

## Supplementary Materials

The following supporting information can be downloaded at: https://www.mdpi.com/article/10.3390/jcm11030847/s1, Table S1: Descriptions of the individual cardiotoxicity risk prediction models, Table S2: Comparison of the patient’s characteristics for the 3 models based on the CTRCD risk category, Table S3: Comparison of patient’s characteristics between our cohort and NSABP 31 CRS and Ezaz model. Available data in the original manuscript are reported, Table S4: Summary of baseline characteristics of the whole cohort (stratified by ESC CTRCD definition), Table S5: Summary of baseline characteristics of the whole cohort (stratified by ASE CTRCD definition), Table S6: Proportion of patients who developed CTRCD with ASE and ESC CTRCD definitions stratified by risk categories for the 3 risk models, Table S7: HFA ICOS proformas, EZAZ and NSABP scores against CREC CTRCD criteria stratified by MUGA versus echocardiography. Percentages reported amongst entire individual risk groups.
