# Supplementary material for: Evaluation of Risk Prediction Models to Identify Cancer Therapeutics Related Cardiac Dysfunction in Women with HER2+ Breast Cancer
Source: J Clin Med. 2022 Feb 5;11(3):847. doi: 10.3390/jcm11030847 (PMC8836544; doi:10.3390/jcm11030847)
Supplement: Supplementary file 1 [file jcm-11-00847-s001.zip › jcm-1558502-supplementary.pdf]

# Evaluation of Risk Prediction Models to Identify Cancer Therapeutics Related Cardiac Dysfunction in Women with HER2+ Breast Cancer

**Supplementary Materials**

Sivisan Suntheralingam MD[1], Chun-Po Steve Fan PhD[2], Oscar Calvillo-Argüelles MD[1,3,4], Husam Abdel-Qadir MD PhD[1,4,5], Eitan Amir, MD, PhD[6], Paaladinesh Thavendiranathan MD SM[1]

**Supplemental Table S1.** Descriptions of the individual cardiotoxicity risk prediction models.

| Risk Scores (Publication Year) | Total (n) | Score Components | Outcome/ Ascertainment | Internal Validation | External validation | Incidence of CTRCD in derviation cohort | Low Risk | Medium Risk | High Risk |
|---|---|---|---|---|---|---|---|---|---|
| **Romond *et al.*,/NSABP-31 (2012)** | 947 | Age<br>LVEF<br>Score = [[7.0 + (0.04 x Age in years) – (0.1 x Baseline LVEF)] x 100] / (4.76) | 7-year Cardiac death or HF symptoms + LVEF decline (>10% decline to <55%, or >5% decline to below lower limit of normal) (MUGA) | Bootstrapping | No | Patients were not categorized into risk groups* | N/A | N/A | N/A |
| **Ezaz *et al.*, (2014)** | 1664 | Age 67-74 = 0 points<br>75-79 = 1 points<br>80-94 = 2 points<br>No chemotherapy = 0 points<br>Non-anthracycline = 2 points<br>Anthracycline = 2 points<br>Diabetes mellitus = 1 point<br>Hypertension = 1 point<br>Coronary artery disease = 2 points<br>Atrial Fibrillation/Flutter = 2 points<br>Renal Failure = 2 points | 3-year HF or cardiomyopathy/ ICD codes from administrative health data | Split sample (n=832) | No | Low risk<br>n = 595 (72%), CTRCD in 86 (14.5%)<br><br>Medium risk<br>n = 195 (23%), CTRCD in 51 (26.2%)<br><br>High risk<br>n = 42 (5%), CTRCD in 18 (42.9%) | Cumulative 0-3 points = Low risk | Cumulative 4-5 points = Medium risk | Cumulative 6+ points = High risk |
| **HFA-ICOS Proformas (2020)** | Expert consensus | Age 65-79 = medium<br>≥80 = high<br>LVEF 50-54% = medium<br>LVEF < 50 % = high<br>Anthracycline before Trastuzumab = medium<br>Remote anthracycline exposure = medium<br>Prior trastuzumab cardiotoxicity = very high<br>Previous radiotherapy to left chest/mediastinum = medium<br>Diabetes mellitus = medium<br>Hypertension = medium<br>Myocardial Infarction/previous coronary revascularization = high<br>Stable angina = high<br>Arrhythmia (Atrial fibrillation/flutter, Ventricular tachycardia/fibrillation) = medium<br>Chronic Kidney Disease = medium<br>Heart failure/cardiomyopathy = very high<br>Severe valvular disease = high | N/A | N/A | No | Suggested incidence of CTRCD:<br>Low risk <2%,<br><br>Medium risk 2-9%,<br><br>High risk 10-19%,<br><br>Very high risk >20% | Low risk = no risk factor OR one medium risk factor | Medium risk = medium risk factors totaling 2-4 points | High Risk = medium risk factors totaling ≥ 5 points OR any high risk factor<br><br>Very high risk = any very high risk factor |

| | | | | |
|---|---|---|---|---|
| | Current/significant smoking history = medium<br>Obesity (BMI > 30) = medium<br>Elevated baseline troponin = medium<br>Elevated baseline BNP/NT-proBNP<br>= medium | | | |

* Note: incidence by risk group was not provided in the original publication, however, when we apply the tertiles identified in our study to the 37 patients with CTRCD in their study of them 5/37 (14%) were in the low risk group (CRS < 50), 14/37 (38%) in the medium risk group (CRS 51-64), and 18/37 (48%) in the high risk group (CRS ≥65). HF, heart failure; LVEF, left ventricular ejection fraction; NSABP-31, National Surgical Adjuvant Breast and Bowel Project B-31; ICD, international classification of diseases; HFA-ICOS, Heart Failure Association of the European Society of Cardiology-International Cardio-Oncology Society; BMI, body mass index; BNP, brain naturetic peptide; NT-proBNP, N-terminal-pro hormone brain natriuretic peptide.

**Supplemental Table S2.** Comparison of the patient's characteristics for the 3 models based on the CTRCD risk category.

| | NSABP-B31 | Ezaz | HFA-ICOS Proforma |
|---|---|---|---|
| *LOW RISK* | N = 223 | N= 577 | N = 193 |
| Age, years ± SD | 47 ± 11 | 51 ± 11 | 49 ± 11 |
| Anthracycline treatment, n (%) | 191 (86) | 514 (89) | 171 (89) |
| Cumulative anthracycline dose, mg/m² ± SD | 239 ± 62 | 266 ± 77 | 255 ± 73 |
| Radiotherapy, n (%) | 210 (94) | 537 (93) | 173 (90) |
| Diabetes, n (%) | 14 (6) | 16 (3) | 1 (0.5) |
| Hypertension, n (%) | 27 (12) | 71 (12) | 4 (2) |
| Dyslipidemia, n (%) | 19 (9) | 59 (10) | 12 (6) |
| Coronary artery disease, n (%) | 7 (3) | 7 (1) | 0 (0) |
| Heart Failure, n (%) | 2 (1) | 3 (0.5) | 1 (0.5) |
| Atrial Fibrillation, n (%) | 4 (2) | 7 (1) | 4 (2) |
| Baseline LVEF, % | 70 ± 7 | 64 ± 7 | 65 ± 7 |
| Cardiotoxicity (CREC), n (%) | 40 (18) | 141 (24) | 30 (16) |
| Cardiotoxicity (ESC), n (%) | 4 (2) | 32 (6) | 8 (4) |
| Cardiotoxicity (ASE), n % | 26 (12) | 93 (16) | 22 (11) |
| *INTERMEDIATE RISK* | N = 204 | N = 45 | N = 404 |
| Age, years ± SD | 54 ± 11 | 61 ± 11 | 53 ± 11 |
| Anthracycline treatment, n (%) | 185 (91) | 39 (86) | 359 (89) |
| Cumulative anthracycline dose, mg/m² ± SD | 263 ± 75 | 239 ± 67 | 271 ± 78 |
| Radiotherapy, n (%) | 189 (93) | 39 (87) | 379 (94) |
| Diabetes, n (%) | 11 (5) | 21 (47) | 30 (7) |
| Hypertension, n (%) | 36 (18) | 33 (73) | 83 (21) |
| Dyslipidemia, n (%) | 30 (15) | 20 (44) | 55 (14) |
| Coronary artery disease, n (%) | 4 (2) | 7 (16) | 8 (2) |
| Heart Failure, n (%) | 2 (1) | 1 (2) | 1 (0.3) |
| Atrial Fibrillation, n (%) | 6 (3) | 3 (7) | 4 (1) |
| Baseline LVEF, n (%) | 64 ± 7 | 64 ± 7 | 64 ± 7 |
| Cardiotoxicity (CREC), n (%) | 50 (25) | 9 (20) | 109 (27) |
| Cardiotoxicity (ESC), n % | 12 (6) | 3 (7) | 26 (6) |
| Cardiotoxicity (ASE), n % | 38 (19) | 8(18) | 71 (18) |
| *HIGH RISK* | N=202 | N = 7 | N= 32 |
| Age, years ± SD | 60 ± 11 | 69 ± 11 | 60 ± 11 |
| Anthracycline treatment, n (%) | 182 (91) | 6 (86) | 29 (91) |
| Cumulative anthracycline dose, mg/m² ± SD | 290 ± 84 | 226 ± 49 | 226 ± 65 |
| Radiotherapy, n (%) | 183 (92) | 5 (71) | 31 (97) |
| Diabetes, n (%) | 16 (8) | 4 (57) | 9 (28) |
| Hypertension, n (%) | 47 (23.5) | 6 (86) | 23 (72) |
| Dyslipidemia, n (%) | 32 (16) | 4 (57) | 15 (47) |
| Coronary artery disease, n (%) | 5 (2.5) | 2 (29) | 8 (25) |
| Heart Failure, n (%) | 1 (1) | 1 (14) | 3 (9) |
| Atrial Fibrillation, n (%) | 2 (1) | 2 (29) | 4 (13) |
| Baseline LVEF | 58 ± 7 | 70 ± 7 | 64 ± 6 |
| Cardiotoxicity (CREC), n (%) | 61 (31) | 1 (14) | 12 (38) |
| Cardiotoxicity (ESC), n % | 19 (10) | 0 (0) | 1 (3) |
| Cardiotoxicity (ASE), n % | 38 (19) | 1(14) | 9 (28) |

SD, standard deviation; NSABP-31, National Surgical Adjuvant Breast and Bowel Project B-31; HFA-ICOS, Heart Failure Association of the European Society of Cardiology-International Cardio-Oncology Society; LVEF, left ventricular ejection fraction; CREC, Cardiac Review and Evaluation Committee; ESC, European Society of Cardiology; ASE, American Society of Echocardiography

**Supplemental Table S3.** Comparison of the patient's characteristics between our cohort and NSABP31-CRS and Ezaz model. Available data in the original manuscript's are reported.

| | **All Patients** | **NSABP** | **EZAZ** |
|---|---|---|---|
| | All Patients<br>N=629 | NSABP<br>N= 947* | EZAZ<br>N=1664 |
| Age, years ± SD | 52.4 ± 10.9 | < 50 years, n= 485<br>50-59 years, n = 311<br>≥ 60 years, n = 148 | 73.6 ± 5.3 |
| Tumor Laterality, n (%) | | | |
| Left | 341 (54) | 354* | - |
| Right | 268 (43) | 590* | - |
| Bilateral | 21 (3) | - | - |
| Stage, n (%) | | | |
| 1 | 127 (20) | - | 446 (27) |
| 2 | 329 (52) | - | 758 (46) |
| 3 | 173 (28) | - | 460 (28) |
| Hormone receptor positive (either ER/PR), n (%) | 413 (66) | - | - |
| Post-Menopausal status, n (%) | 309 (49) | - | - |
| Surgery (mastectomy or lumpectomy), n (%) | 622 (99) | - | 1664 (100) |
| Anthracycline received, n (%) | 568 (90) | - | 597 (36) |
| Cumulative anthracycline, $mg/m^2$ ± SD | 272.4 ± 76 | - | - |
| Radiotherapy, n (%) | 493 (78) | - | 949 (57) |
| Diabetes, n (%) | 44 (7) | 36^ | 325 (20) |
| Hypertension, n (%) | 110 (18) | 193^ | 1002 (60) |
| Dyslipidemia, n (%) | 83 (13) | 70^ | 981 (59) |
| Smoker (current), n (%) | 46 (7) | 374* | - |
| Smoker (Ex), n (%) | 122 (19) | - | - |
| At least 1 CVRF, n (%) | 402 (64) | - | 590 (35) |
| Coronary artery disease, n (%) | 16 (3) | - | 60 (4) |
| Heart Failure, n (%) | 5 (1) | - | - |
| Atrial Fibrillation, n (%) | 12 (2) | - | 90 (5) |
| Baseline LVEF, % ± SD | 64 ± 7 | ≥ 65%, n= 351<br>55-64%, n=473<br>50-54%, n=111 | - |
| Imaging Modality, n (%) | | | |
| MUGA | 440 (70) | - | - |
| Echo | 189 (30) | - | - |

(-) indicate this data was not reported in the original study
* tables in original data do not add up to 947 patients and so there is some missing data from the original studies that was recorded and overall percentages are not reported
^ these were listed as medications for these co-morbidities and there could be under-representation of these co-morbidities

**Supplemental Table S4.** Summary of baseline characteristics of the whole cohort (stratified by ESC CTRCD definition)

| | **CTRCD**<br>**N= 35** | **No CTRCD**<br>**N= 594** | **P Value** |
|---|---|---|---|
| Age, years ± SD | 53 ± 11 | 52 ± 11 | 0.5778 |
| Anthracycline treatment, n (%) | 31 (89) | 527 (89) | 0.9783 |
| Cumulative anthracycline dose mg/m$^2$ ± SD | 262 ± 79 | 263 ± 76 | 0.9449 |
| Radiotherapy, n(%) | 28 (80) | 465 (78) | 0.8104 |
| Diabetes, n (%) | 2 (6) | 38 (6) | 0.8721 |
| Hypertension, n (%) | 5 (14) | 104 (18) | 0.6245 |
| Dyslipidemia, n (%) | 5 (14) | 78 (13) | 0.8445 |
| Smoker (current) | 2 (6) | 43 (7) | 0.7338 |
| Smoker (Ex) | 5 (14) | 117 (20) | 0.4314 |
| At least 1 CVRF, n (%) | 15 (43) | 271 (46) | 0.7495 |
| Coronary artery disease, n (%) | 0 (0) | 17 (3) | 0.6161 |
| Heart Failure, n (%) | 0 (0) | 6 (1) | 1.000 |
| Atrial Fibrillation, n (%) | 1 (3) | 11 (2) | 0.6727 |
| Baseline LVEF, % | 56 ± 7 | 64 ± 6 | **0.0201** |
| *Imaging Modality, n (%)* | | | 0.3497 |
| MUGA | 27 (77) | 414 (70) | |
| Echo | 8 (23) | 180 (30) | |

SD, standard deviation; CRVF, cardiovascular risk factor; LVEF, left ventricular ejection fraction; ESC, European Society of Cardiology; MUGA, multi-gated acquisition; Echo, echocardiography.

**Supplemental Table S5.** Summary of baseline characteristics of the whole cohort (stratified by ASE CTRCD definition)

| | **CTRCD N= 102** | **No CTRCD N= 527** | **P Value** |
|---|---|---|---|
| Age, years ± SD | 54 ± 11 | 52 ± 11 | 0.3912 |
| Anthracycline treatment, n (%) | 92 (90) | 468 (89) | 0.6806 |
| Cumulative anthracycline dose mg/m² ± SD | 275 ± 81 | 262 ± 76 | 0.1416 |
| Radiotherapy, n(%) | 90 (88) | 413 (78) | **0.0223** |
| Diabetes, n (%) | 5 (5) | 32 (6) | 0.6457 |
| Hypertension, n (%) | 22 (22) | 88 (17) | 0.2359 |
| Dyslipidemia, n (%) | 16 (16) | 67 (13) | 0.4168 |
| Smoker (current) | 8 (8) | 38 (7) | 0.8223 |
| Smoker (Ex) | 17 (17) | 105 (20) | 0.4463 |
| At least 1 CVRF, n (%) | 25 (25) | 143 (27) | 0.6049 |
| Coronary artery disease, n (%) | 2 (2) | 14 (3) | 0.6829 |
| Heart Failure, n (%) | 2 (2) | 3 (1) | 0.1475 |
| Atrial Fibrillation, n (%) | 5 (5) | 10 (2) | 0.0687 |
| Baseline LVEF, % | 63 ± 7 | 65 ± 7 | **<0.001** |
| *Imaging Modality, n (%)* | | | **0.049** |
| MUGA | 63 (62) | 377 (72) | |
| Echo | 39 (38) | 150 (28) | |

SD, standard deviation; CRVF, cardiovascular risk factor; LVEF, left ventricular ejection fraction; ASE, American Society of Echocardiography; MUGA, multi-gated acquisition; Echo, echocardiography.

**Supplemental Table S6.** Proportion of patients who developed CTRCD with ASE and ESC CTRCD definitions stratified by risk categories for the 3 risk models.

| **EZAZ** | **Total no. of patients** | **ASE CTRCD N = 102** | **ESC CTRCD N = 35** |
|---|---|---|---|
| **Risk categories** | | | |
| Low, n (%) | 577 | 93 (16) | 32 (6) |
| Medium, n (%) | 45 | 8 (18) | 3 (7) |
| High/, n (%) | 7 | 1 (14) | 0 (0) |
| P value† | - | 0.949 | - |

| **NSABP-31 CRS** | **Total no. of patients** | **ASE CTRCD N = 102** | **ESC CTRCD N = 35** |
|---|---|---|---|
| **Risk categories** | | | |
| Low, n (%) | 223 | 26 (12) | 4 (2) |
| Medium, n (%) | 204 | 38 (19) | 12 (6) |
| High, n (%) | 202 | 38 (19) | 19 (10) |
| P value† | - | 0.067 | **0.003** |

| **HFA-ICOS** | **Total no. of patients** | **ASE CTRCD N = 102** | **ESC CTRCD N = 35** |
|---|---|---|---|
| **Risk categories** | | | |
| Low, n (%) | 193 | 22 (11) | 8 (4) |
| Medium, n (%) | 404 | 71 (18) | 26 (6) |
| High, n (%) | 27 | 6 (22) | 1 (4) |
| Very high, n (%) | 5 | 3 (60) | 0 (0) |
| P value† | | **0.001** | - |

*p values represent comparison between the ASE and ESC cardiotoxicity criteria, some p values not reported because of 0 values; †p value represents comparison between the 3 risk categories for that specific score and the respective CTRCD definition. CTRCD refers to cancer therapeutics associated cardiac dysfunction; NSABP-31, National Surgical Adjuvant Breast and Bowel Project B-31; HFA-ICOS, Heart Failure Association of the European Society of Cardiology-International Cardio-Oncology Society; ESC, European Society of Cardiology; ASE, American Society of Echocardiography

**Supplemental Table S7.** Incidence of CTRCD (CREC criteria) with the thre models tratified by MUGA versus echocardiography. Percentages reported amongst entire individual risk groups.

| **EZAZ** | **Total no. of patients** | **Total MUGA (n = 440)** | **MUGA CREC CTRCD (n = 93)** | **Total ECHO (n = 58)** | **ECHO CREC CTRCD (n = 58)** | **P Value** |
|---|---|---|---|---|---|---|
| **Risk categories** | | | | | | |
| Low, n (%) | 577 | 397 (69) | 86 (22) | 180 (31) | 55 (31) | **0.021** |
| Medium, n (%) | 45 | 36 (80) | 6 (17) | 9 (20) | 3 (33) | 0.264 |
| High, n (%) | 7 | 7 (100) | 1 (14) | 0 (0) | 0 (0) | - |

| **NSABP-31** | **Total no. of patients** | **Total MUGA (n = 440)** | **MUGA CREC CTRCD (n = 93)** | **Total ECHO (n = 58)** | **ECHO CREC CTRCD (n = 58)** | **P Value** |
|---|---|---|---|---|---|---|
| **Risk categories** | | | | | | |
| Low, n (%) | 223 | 191 (86) | 29 (15) | 32 (14) | 11 (34) | **0.001** |
| Medium, n (%) | 204 | 140 (69) | 32 (23) | 64 (31) | 18 (28) | 0.417 |
| High, n (%) | 202 | 109 (54) | 32 (30) | 93 (46) | 29 (31) | 0.778 |

| **HFA-ICOS** | **Total no. of patients** | **Total MUGA (n = 440)** | **MUGA CREC CTRCD (n = 93)** | **Total ECHO (n = 58)** | **ECHO CREC CTRCD (n = 58)** | **P Value** |
|---|---|---|---|---|---|---|
| **Risk categories** | | | | | | |
| Low, n (%) | 193 | 146 (76) | 21 (14) | 47 (24) | 9 (19) | 0.432 |
| Medium, n(%) | 404 | 266 (66) | 62 (23) | 138 (34) | 47 (34) | **0.021** |
| High, n (%) | 27 | 23 (85) | 8 (35) | 4 (15) | 2 (50) | 0.561 |
| Very high, n (%) | 5 | 5 (100) | 2 (40) | 0 (0) | 0 (0) | - |

CTRCD, cancer therapeutics associated cardiac dysfunction; CREC, Cardiac Review and Evaluation Committee; MUGA, multi-gated acquisition; Echo, echocardiography**;** NSABP-31, National Surgical Adjuvant Breast and Bowel Project B-31; HFA-ICOS, Heart Failure Association of the European Society of Cardiology-International Cardio-Oncology Society; ESC, European Society of Cardiology; ASE, American Society of Echocardiography. Some p values not reported because of 0 values.
